# Population pharmacokinetics of Artemether and dihydroartemisinin in pregnant women with uncomplicated *Plasmodium falciparum* malaria in Uganda

**DOI:** 10.1186/1475-2875-11-293

**Published:** 2012-08-22

**Authors:** Joel Tarning, Frank Kloprogge, Patrice Piola, Mehul Dhorda, Sulaiman Muwanga, Eleanor Turyakira, Nitra Nuengchamnong, François Nosten, Nicholas PJ Day, Nicholas J White, Philippe J Guerin, Niklas Lindegardh

**Affiliations:** 1 Centre for Tropical Medicine, Nuffield Department of Clinical Medicine, University of Oxford, Oxford, UK; 2 Mahidol-Oxford Tropical Medicine Research Unit, Faculty of Tropical Medicine, Mahidol University, Bangkok, Thailand; 3 Epicentre, Paris, France; 4 Epicentre, Mbarara, Uganda; 5 Center for Vaccine Development, University of Maryland School of Medicine, Baltimore, MD, USA; 6 Mbarara, University of Science & Technology, Mbarara, Uganda; 7 Shoklo Malaria Research Unit, Mae Sot, Thailand

**Keywords:** Non-linear mixed effects modeling, Pharmacokinetics, Artemether, Dihydroartemisinin, Pregnancy, Malaria

## Abstract

**Background:**

Malaria in pregnancy increases the risk of maternal anemia, abortion and low birth weight. Approximately 85.3 million pregnancies occur annually in areas with *Plasmodium falciparum* transmission. Pregnancy has been reported to alter the pharmacokinetic properties of many anti-malarial drugs. Reduced drug exposure increases the risk of treatment failure. The objective of this study was to evaluate the population pharmacokinetic properties of artemether and its active metabolite dihydroartemisinin in pregnant women with uncomplicated *P. falciparum* malaria in Uganda.

**Methods:**

Twenty-one women with uncomplicated *P. falciparum* malaria in the second and third trimesters of pregnancy received the fixed oral combination of 80 mg artemether and 480 mg lumefantrine twice daily for three days. Artemether and dihydroartemisinin plasma concentrations after the last dose administration were quantified using liquid chromatography coupled to tandem mass-spectroscopy. A simultaneous drug-metabolite population pharmacokinetic model for artemether and dihydroartemisinin was developed taking into account different disposition, absorption, error and covariate models. A separate modeling approach and a non-compartmental analysis (NCA) were also performed to enable a comparison with literature values and different modeling strategies.

**Results:**

The treatment was well tolerated and there were no cases of recurrent malaria. A flexible absorption model with sequential zero-order and transit-compartment absorption followed by a simultaneous one-compartment disposition model for both artemether and dihydroartemisinin provided the best fit to the data. Artemether and dihydroartemisinin exposure was lower than that reported in non-pregnant populations. An approximately four-fold higher apparent volume of distribution for dihydroartemisinin was obtained by non-compartmental analysis and separate modeling compared to that from simultaneous modeling of the drug and metabolite. This highlights a potential pitfall when analyzing drug/metabolite data with traditional approaches.

**Conclusion:**

The population pharmacokinetic properties of artemether and dihydroartemisinin, in pregnant women with uncomplicated *P. falciparum* malaria in Uganda, were described satisfactorily by a simultaneous drug-metabolite model without covariates. Concentrations of artemether and its metabolite dihydroartemisinin were relatively low in pregnancy compared to literature data. However, this should be interpreted with caution considered the limited literature available. Further studies in larger series are urgently needed for this vulnerable group.

## Background

Malaria is a major cause of morbidity and mortality in pregnancy [1]. An estimated 85.3 million pregnancies occurred in 2007 in areas with *Plasmodium falciparum* transmission [2]. The susceptibility to malaria is increased during pregnancy as a result of immunological and hormonal changes [3,4]. *P. falciparum* malaria in pregnancy is associated with increased anaemia and a higher risk of severe malaria and death compared to a non-pregnant adult population [5]. Parasitized erythrocytes accumulate in the placenta [3,6,7]. Malaria reduces birth weight through intrauterine growth retardation and preterm delivery [8].

Pregnancy has been reported to alter the pharmacokinetic properties of many anti-malarial drugs. Lower drug exposure in pregnant women has previously been reported for artemether/dihydroartemisinin [9], artesunate/dihydroartemisinin [10], dihydroartemisinin [11], lumefantrine [12], atovaquone [13], proguanil [13], sulphadoxine [14] and pyrimethamine [15]. This may increase the risk of treatment failure, particularly when immune responses to malaria are suppressed during pregnancy. In contrast some studies show similar (e g, pyrimethamine, amodiaquine and desethylamodiaquine) or higher (e g, pyrimethamine, sulphadoxine and mefloquine) anti-malarial drug exposure in pregnant women compared to the non-pregnant adult patient population [14-20]. Different pharmacokinetic analytical methodologies, such as non-compartmental analysis (NCA), separate and simultaneous population pharmacokinetic analysis, have been employed which further complicates the interpretation. Comparison of parameter estimates obtained with different methodologies should be performed with caution.

Artemisinin-based combination therapy (ACT) is recommended as first-line treatment by the World Health Organization (WHO) for uncomplicated *P. falciparum* malaria [1]. The fixed oral combination of artemether and lumefantrine is one of the most widely used ACTs and gives high cure rates (>95%) and good tolerability in children and adults with uncomplicated *P. falciparum* malaria [21-23]. However, unacceptably low cure rates were reported for pregnant women (n = 124) on the north-west border of Thailand (PCR-corrected cure rate of 82.0% (95% CI. 74.8-89.3) at delivery or day 42 if later) with a standard fixed combination explained by low drug concentrations in late pregnancy [12,24]. On the other hand, high efficacy (PCR-corrected cure rate of 98.2% (95% CI. 93.5-99.7) at delivery or day 42 if later) was reported in pregnant women in Uganda (n = 152) when treated with a standard regimen of artemether and lumefantrine [25]. Transmission, and therefore immunity, is substantially higher in Uganda than in Thailand, but pharmacokinetic differences may also contribute to these findings.

The objective of this study was to characterize the population pharmacokinetic properties of artemether and its metabolite dihydroartemisinin in pregnant women with uncomplicated *P. falciparum* malaria in Uganda.

## Methods

### Study design

This pharmacokinetic study was nested into a larger efficacy study conducted in the Mbarara National Referral Hospital (MNRH) antenatal clinic (ANC) in Uganda. Full clinical details are reported elsewhere [25]. Ethical approval was obtained from the Mbarara University Faculty of Medicine Research and Ethics Committee, the Mbarara University Institutional Ethics Committee, the Uganda National Council for Science and Technology (ethics committee) and the de Protection des Personnes de St. Germain en Laye, lle de France XI. The trial was registered at ClinicalTrials.gov (NCT00495508). The patients were recruited from March to September 2008. Inclusion criteria were *P. falciparum* mixed- or mono-infection (detected by microscopy), residence in the Mbarara municipality (radius 15 km from MNRH) and an estimated gestation age (EGA) of at least 13 weeks. Exclusion criteria were *P. falciparum* parasitaemia above 250,000 parasite/μL, severe anaemia (Hb <7 g/dL), signs or symptoms of severe malaria requiring parental treatment, known allergy to artemisinin derivates, lumefantrine or quinine, previous participation in the efficacy study or inability to comply with the specified follow-up schedule. Patients were enrolled if they fulfilled all of the inclusion criteria, none of the exclusion criteria, and if written informed consent was obtained. The presented population pharmacokinetic analysis was conducted using the dense artemether/dihydroartemisinin samples.

### Dose regimen and blood samples

Four tablets of the fixed oral combination of artemether and lumefantrine (Coartem® Novartis Pharma AG, Basel, Switzerland; each tablet contained 20 mg artemether and 120 mg lumefantrine) were administered twice daily for three days (0, 8, 24, 36, 48 and 60 hours) with 200 mL of milk tea at each dose to optimize the oral bio-availability of lumefantrine [26]. A full replacement dose was given if the dose was vomited within 30 min and a half replacement dose was given if the dose was vomited between 30 min and one hour. The patient was withdrawn from the study and treated with rescue treatment if the replacement dose was vomited again within 30 min. Venous blood samples (2 mL) were drawn from a cannula into heparinized tubes at 0, 0.25, 0.5, 0.75, 1, 1.25, 1.5, 1.75, 2, 2.5, 3, 4, 6, 8, and 10 hours after the last dose.

### Drug analysis

Blood samples were centrifuged at 1,400 g for 5 min and plasma was stored at -70°C until analysis. Plasma samples were shipped on dry ice to MORU Clinical Pharmacology Laboratory, Bangkok, Thailand for drug quantification. Quantification of artemether and dihydroartemisinin was performed by a previously published method [27]. Artemether and dihydroartemisinin and their stable isotope labeled internal standards were extracted from plasma using solid phase extraction (HLB u-elution SPE 96-well plate, Waters, USA) separated and quantified by liquid chromatography (Agilent 1200 system, Agilent Technologies, USA) coupled to positive electro spray tandem mass spectroscopy (API 5000 triple quadrupole, Applied Bios stems/MDS SCIEX, USA). To ensure precision and accuracy during quantification, triplicates of quality control samples at three concentrations; 3.46 ng/ml, 36.0 ng/ml and 375 ng/ml for both artemether and dihydroartemisinin were analyzed with every batch. The overall accuracy (i e, relative standard deviation) was less than 5.4%. The limit of detection (LOD) was set to 0.5 ng/mL and the lower limit of quantification (LLOQ) was set to 1.43 ng/mL for both compounds. The MORU laboratory is a participant in the QA/QC programmed supported by the Worldwide Antimalarial resistance Network (WWARN).

### Compartmental analysis

Artemether and dihydroartemisinin dose and plasma concentrations were converted into molar units and modeled as the natural logarithm of the molar plasma concentrations. Modeling and simulation was performed on a Windows XP operating system (Microsoft Corporation, Seattle, WA, USA) with a G95 Fortran compiler (Free Software Foundation, Boston, MA, USA) using NONMEM v.7.1 (ICON Development Solutions, Ellicott City, MD, USA). ADVAN5, TRANS1 and the first order conditional estimation method with interaction was used during model building [28]. Post-processing and automation was performed using Pearl-Speaks-NONMEM (PsN) v. 3.2.12 [29,30], Census v. 1.2b2 [31], Xpose v. 4 [32] and R v. 2.10.1 (The R Foundation for Statistical Computing).

The objective function value (OFV) computed as minus twice the log likelihood of the data, physiological plausibility and goodness-of-fit diagnostics were used to evaluate competing models during the model building process. A reduction in OFV of 3.84 or more was considered a significant (p = 0.05) improvement after the introduction of one new parameter (one degree of freedom).

Pharmacokinetic properties of artemether and dihydroartemisinin were modeled both separately and simultaneously using a one-compartment disposition model with first-order absorption and elimination for both artemether and dihydroartemisinin. Complete conversion of artemether into dihydroartemisinin was assumed for all modeling approaches [33,34]. The population pharmacokinetic models were parameterized using a first-order absorption rate constant (ka), artemether elimination clearance (CL_ARM_/F), apparent artemether volume of distribution (V_ARM_/F), dihydroartemisinin elimination clearance (CL_DHA_/F), and apparent dihydroartemisinin volume of distribution (V_DHA_/F). Inter-individual variability (IIV) was implemented exponentially for all parameters.

The simultaneous population pharmacokinetic base model was optimized further in order to describe accurately the pharmacokinetic properties of artemether and dihydroartemisinin. The implementation of relative bioavailability was investigated followed by addition of one and two peripheral distribution compartments for both artemether and dihydroartemisinin. Enterohepatic recirculation of artemether was evaluated by applying a model event time (MPAST) to the rate constant from a peripheral compartment to the central compartment. This generated continuous flow from the central compartment to a hypothetical biliary compartment and a time-dependent backflow to the gut compartment mimicking the enterohepatic circulation. A semi-mechanistic liver model structure described by Gordi *et al* was also applied to the data in order to describe partial pre-systemic conversion of artemether into dihydroartemisinin [35].

Several absorption models were evaluated in combination with the most appropriate body structure; first-order, parallel first-order, zero-order, parallel first- and zero-order and zero order absorption followed by first-order absorption with and without lag-time. An alternative way to describe partial pre-systemic conversion of artemether into dihydroartemisinin was also considered by an estimated ratio of dual first order absorption of artemether and dihydroartemisinin. A transit-compartment absorption model with an individually estimated number of transit compartments was tried and compared to a less flexible transit-compartment absorption model where the number of transit-compartments (1-10) was evaluated and fixed for the population. A semi-mechanistic transit-compartment absorption model was also evaluated combining zero-order dissolution of the drug before drug absorption via a fixed number of transit compartments. A correlation matrix of more than 50% between variability components was considered as a significant contribution. Additive, proportional and intercept-slope error models were evaluated to explain residual random variability of artemether and dihydroartemisinin. Separate and combined error models for artemether and dihydroartemisinin were evaluated.

Different methodologies to avoid bias in parameter estimates caused by multiple samples being below the limit of quantification (BLOQ) were evaluated [36-38]. BLOQ data were imputed by a fixed concentration at LLOQ/2 or modeled as censored data using the M3 method [36-38] in combination with Laplacian estimation.

All covariates (Table 1) were screened by adding them individually on each of the pharmacokinetic parameters in the model using a linear and an exponential relationship. Significant covariates (p < 0.05, ΔOFV > 3.84) that were considered physiologically plausible were evaluated through forward addition and backward elimination covariate selection (SCM, [29,39]). A p-value of 0.05 was used in the forward step and a p-value of 0.01 (ΔOFV > 6.63) was considered significant for retaining a covariate in the model during the backward elimination. Body weight was also evaluated as an allometric function on all clearance and volume parameters. A model with estimated age of gestation as a covariate on CL_ARM_, V_ARM_, CL_DHA_, V_DHA_, and MTT in a linear relationship was evaluated for a full-covariate model approach. 

**Table 1:** Demographic information of the study population

	**Mean ± S.D.**	**Median (range)**
Number of patients	21	
Total artemether dose (mg/kg)	8.46 ± 1.22	8.73 [5.46-9.80]
Total number of samples	316	
Sample size (samples/patient)	15.0 ± 0.805	15 [12-16]
*covariates*		
Body weight (kg)	58.1 ± 10.1	55 [49-88]
Age (years)	21.4 ± 4.28	21 [16-35]
Gestational age (weeks)	25.8 ± 7.77	27 [13-36]
Haemoglobin (g/dL)	11.1 ± 1.72	11.3 [7.6-14.6]
Red blood cell count (10^6^ cells/cmm)	3.74 ± 0.629	3.71 (2.37-4.79)
Haematocrit (%)	33.7 ± 5.14	34.0 (23.2-44.5)
Neutrophils (counts/μL)	2.73 ± 0.802	2.75 (1.14-4.13)
Eosinophils (counts/μL)	0.130 ± 0.148	0.0700 [0.0200-0.570]
Basophils (counts/μL)	0.0280 ± 0.0140	0.0200 [0.0100-0.0600]
Lymphocytes (counts/μL)	2.08 ± 0.667	1.98 [1.12-3.51]
Monocytes (counts/μL)	0.590 ± 0.214	0.550 [0.260-1.00]
Platelets (10^3^/cmm)	166 ± 62.0	167 [64-285]
Alanine aminotransferase (IU/L)	16.1 ± 6.77	14.0 [5.00-35.0]
Creatinine (mg/dl)	0.481 ± 0.103	0.470 [0.330-0.660]
Bilirubin (mg/dl)	1.24 ± 1.10	0.910 [0.560-5.53]
Diastolic blood pressure (mmHg)	60.1 ± 6.20	60.0 [46.0-75.0]
Temperature (°C)	36.8 ± 0.747	36.7 [36.0-38.5]
*P. falciparum* parasitaemia (parasites/μL)	10900 ± 32000	1570 [88.0-148000]

Eta and epsilon shrinkage was calculated to assess the reliability of individual parameter estimates and goodness-of-fit diagnostics [40]. A non-parametric bootstrap of 1,000 datasets was performed in order to calculate non-parametric confidence intervals. The predictive power of the model was examined by visual and numerical predictive checks, using 2,000 simulations of each individual plasma concentration series [41]. The 95% confidence intervals of the simulated 5^th^, 50^th^ and 95^th^ percentile were overlaid with the 5^th^, 50^th^ and 95^th^ percentile of the observed data.

### Non-compartmental analysis

Individual concentration-time data were analyzed with NCA using WinNonlin v. 5.3 (Pharsight Corporation, California, USA). Complete *in vivo* conversion of artemether into dihydroartemisinin was assumed [42]. The dose of dihydroartemisinin was calculated using the relative difference in molecular weight of artemether and dihydroartemisinin [dose_dihydroartemisinin_ = dose_artemether_ × (MW_dihydroartemisinin_: 284.3 g/mol)/(MW_artemether_: 298.4 g/mol)]. Total exposure up to the last measured concentration (AUC_0-LAST_) was calculated using the linear trapezoidal method for ascending concentrations and the logarithmic trapezoidal method for descending concentrations. Extrapolation from the last observed concentration was performed using C_LAST_/λ_Z_ for each individual subject. A log-linear regression was used to estimate the terminal elimination half-life using the observed concentrations in the terminal elimination phase. Maximum concentration (C_MAX_) and time to C_MAX_ (T_MAX_) were taken directly from the observed data. Standard procedures in WinNonlin were used to compute the individual values for apparent volume of distribution (V_Z_/F) and oral elimination clearance (CL/F).

Pharmacokinetic parameter estimates from the NCA and separate modeling were compared to that produced by a simultaneous modeling strategy. Small but systematic differences in individual parameter estimates might result in significant differences between methodologies, when using paired tests, but will be of no clinical relevance. A student *t*-test was therefore used to compare logarithmically transformed parameter estimates between two methodologies. ANOVA with regression analysis was performed to compare logarithmically transformed parameter estimates between more than two methodologies.

## Results

### Demographic information

Twenty-one (21) pregnant women in their second and third trimesters from Uganda were enrolled in the study (Table 1). The treatment was well tolerated and no cases of vomiting or recurrent malaria infections were recorded.

### Pharmacokinetic analysis

#### Compartmental analysis of artemether and dihydroartemisinin

A zero-order absorption followed by transit compartment absorption described the artemether and dihydroartemisinin absorption better than all other absorption models (ΔOFV > -71). The administered drug disintegrates in the gut, resulting in a continuous drug supply, described by a zero-order process followed by transit absorption of drug into the systemic circulation. Six transit compartments were sufficient to describe the data. Other absorption models were not better and/or produced unreliable parameter estimates (RSE > 50%). The implementation of a pre-systemic artemether elimination pathway in the model (ΔOFV = -12.5) was only possible in combination with a two-compartment disposition of artemether and the M3 method, but this resulted in an unrealistic artemether elimination half-life of 48.8 h [46.7-53.1] so this model structure was not considered as superior.

**Figure 1:**
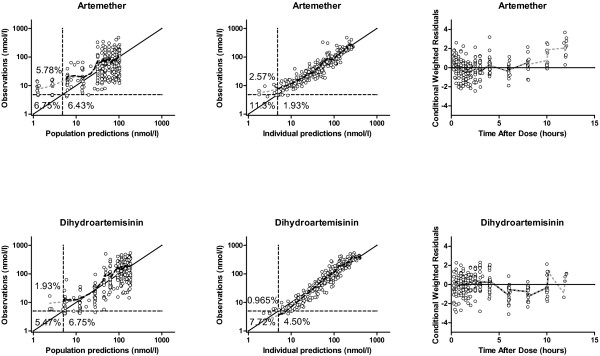
**Artemether and dihydroartemisinin goodness-of-fit.** The solid black line represents the line of identity, the local polynomial regression fitting for observations predicted above LLOQ is represented by the dashed black line and the local polynomial regression fitting for all observations is represented by the grey dashed line. The horizontal and vertical dashed black lines represent the lower limit of quantification (LLOQ). Clinical observations are represented by the black circles. Percentages mentioned in the diagnostic plots represent the percentages of the total amount of data in the particular subset.

A simultaneous one-compartment drug-metabolite model best described the disposition pharmacokinetics of artemether and dihydroartemisinin. Goodness-of-fit diagnostics of the final model showed an adequate description of observed data (Figure 1). The under-prediction of low artemether and dihydroartemisinin concentrations is a direct consequence of a high proportion of data below the LLOQ. The goodness-of-fit diagnostics in the present study (Figure 1) suggested that a two-compartment disposition model for dihydroartemisinin might be a better description of the data but the addition of a peripheral compartment for dihydroartemisinin did not improve the model fit (ΔOFV = 0.003). Another study in children (one to 10 years old) with uncomplicated *P. falciparum* malaria in Tanzania was best described by a simultaneous artemether-dihydroartemisinin model consisting of two- and one-disposition compartments for artemether and dihydroartemisinin, respectively [33]. The addition of a peripheral compartment for artemether improved the model fit (ΔOFV = -14.8) but could not be retained due to a combination of poor precision (RSE > 30%) in additional parameters and misspecification of censored data. Implementation of the M3 method solved the misspecification of censored data but poor parameter precision (RSE > 30%) remained. Incorporation of inter-individual variability in the relative bioavailability significantly improved the model fit (ΔOFV = -133) due to variable absorption of artemether. 

A combined additive error model for both the drug and the metabolite was sufficient to describe the random residual variability in the data. This is not unexpected since artemether and dihydroartemisinin plasma samples were obtained from the same blood sample and concentrations were quantified using a simultaneous bioanalytical method.

In the final model, the absorption rate constant was set to be identical to the rate constant between transit compartments because of the poor precision of the absorption rate constant (RSE > 50%). IIV for the distribution volume of artemether and dihydroartemisinin were fixed to zero because of poor precision (RSE > 50%). Incorporation of relative bioavailability should theoretically decorrelate pharmacokinetic parameters (i e, clearance and volume parameters) within a patient. As expected, variability components between these parameters were not correlated (<50% correlation) in the final model.

**Figure 2:**
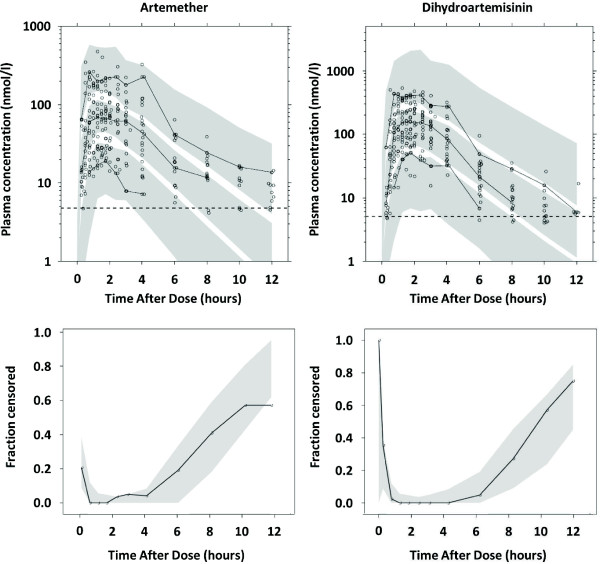
**Visual Predictive Check of plasma artemether and dihydroartemisinin concentrations.** Upper panel: open circles represent the observed data, the solid lines the 5^th^, 50^th^ and 95^th^ percentiles of the observed data and the shaded area the 95% confidence intervals of the 5^th^, 50^th^ and 95^th^ percentiles of the simulated plasma concentrations (nmol/L). The limit of quantification is represented by the black dashed line. Lower panel: the shaded area represents the simulated 95% confidence intervals for the fraction of BQL data. The black solid line represents the observed fraction of BQL data.

The relatively short half-life of artemether and dihydroartemisinin can cause a bias in parameter estimates because a large proportion of concentration measurements below the LLOQ (i.e. 14.9% and 47.6% of artemether and 13.7% and 33.3% of dihydroartemisinin samples were below the LLOQ in total and at 10 hours after dose, respectively). Coding BLOQ data as missing data performed well with no trends of over- or under-predicting BLOQ data (Figure 2). Incorporation of the M3 method or imputing BLOQ data with LLOQ/2 resulted only in minor improvements in the visual diagnostics. The M3 and LLOQ/2 approach resulted in much higher condition numbers compared to the conventional method of coding BLOQ data as missing data, which implies that these models are less robust. BLOQ data were therefore coded as missing data in the final model.

**Figure 3:**
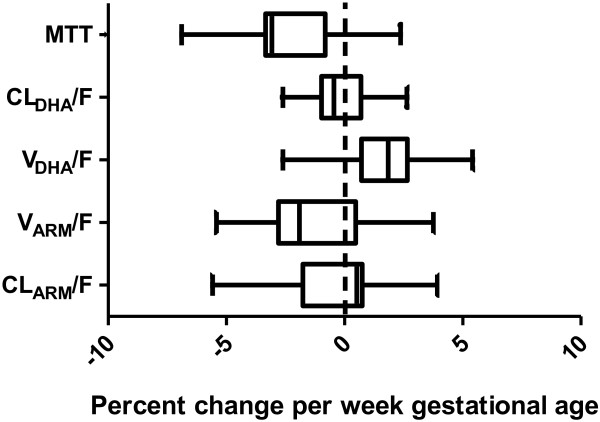
**Boxplots (2.5 - 97.5 percentiles) visualising the effect of estimated gestational age on pharmacokinetic parameters.** Mean transit time (MTT), apparent volume of distribution dihydroartemisinin (V_DHA_/F), elimination clearance (CL_DHA_/F), apparent volume of distribution artemether (V_ARM_/F) and elimination clearance artemether (CL_ARM_/F) from 250 bootstrap runs.

There were no statistically significant covariates in this study. Artemether is predominantly metabolized by cytochrome 3A4 [43] and dihydroartemisinin by UGT 1A9 and UGT 2B7 [44]. Both the hepatic and intestinal CYP 3A4 activities have been reported to be induced during pregnancy compared with post-partum women [45,46]. Between the second and third trimester of pregnancy no difference in CYP 3A4 activity has been observed [45]. This might explain why no covariate effect of estimated age of gestation could be found on artemether elimination clearance in this study. DHA is eliminated via glucuronidation and limited evidence suggests higher UGT 1A9 and UGT2B7 activities at the time of delivery compared with non-pregnant women [47,48]. However, no covariate effect of estimated age of gestation was found on dihydroartemisinin elimination clearance either. This might indicate that there is no difference in UGT 1A9 and UGT2B7 activity between the second and third trimester. As there was no non-pregnant control group, pregnancy could not be evaluated as a categorical covariate in this study. A full covariate approach was applied to enable a visual inspection of the estimated age of gestation effect on CL_ARM_/F, V_ARM_/F, CL_DHA_/F, V_DHA_/F and MTT (Figure 3). The covariate effect was distributed with a certainty of 95% between -7.0% and 5.5% change in parameter estimate per estimated age of gestation in weeks, confirming the absence of significant covariate effects from estimated age of gestation in the studied population. 

The numerical predictive check of the final model computed 1.11% (95% CI. 0.74 to 11.11%) and 2.96% (95% CI, 0.74-11.85%) of the observed artemether concentrations below and above the 90% prediction interval, respectively. For dihydroartemisinin 0% and 0.37% (95% CI. 0.37-12.45%) of observations were calculated below and above the 90% prediction interval, respectively. This indicated an over-prediction of the variability from both the drug and the metabolite. This was a result of problems with fitting the erratic absorption phase and a relatively small study population (Figure 2). No cases of vomiting were reported nor were there other possible explanations for the observed absorption characteristics such as concomitant therapy. Similar erratic absorption profiles have been reported previously in healthy volunteers [49] and similar over prediction was reported in children with uncomplicated malaria in Tanzania [33].

The central tendencies of the concentration-time profiles are predicted adequately and population parameter estimates were robust but showed large inter-individual variability as indicated by the predictive checks (Table 2 and Figure 2). All shrinkage estimates were below 20% indicating the reliability of the individual parameter estimates.

**Table 2:** Parameter estimates for the final simultaneous artemether and dihydroartemisinin model

**Parameter**	**Population estimate**^**a**^	**95% CI**^**b**^	**IIV[%CV]**^**a**^	**95% CI**^**b**^
	**(% RSE)**^**b**^		**(% RSE)**^**b**^	
CL_ARM_/F (L/hr)	875 (18.7)	625-1280	28.0 (47.6)	12.0-37.8
V_ARM_/F (L)	2160 (17.4)	1620-3100	-	-
CL_DHA_/F (L/hr)	468 (10.2)	387-588	90.4 (39.0)	40.5-126
V_DHA_/F (L)	57.1 (20.1)	41.7-88.8	-	-
MTT (hr)	0.274 (19.4)	0.174-0.378	75.2 (39.6)	41.4-121
DUR (hr)	0.687 (25.5)	0.380-1.14	151 (24.1)	90.6-209
F	1 (*fixed*)	-	85.5 (24.8)	53.2-108
No. of transit compartments	6 (*fixed*)	-	-	-
σ	0.166 (6.87)	0.130-0.221	23.1 (51.7)	8.35-35.2
**Post-hoc estimates parameters**^**c**^	**Artemether Median (range)**	**Dihydroartemisinin Median (range)**		
AUC_60h-∞_ (hr × ng/mL)	111 (16.2-317)	167 (55.3-437)		
C_MAX_ (ng/mL)	32.9 (7.5-82.8)	45.2 (14.1-114)		
T_MAX_ (hr)	1.16 (0.65-3.81)	1.37 (0.82-3.89)		

^a^Population mean values and inter-individual variability (IIV) estimated by NONMEM. IIV is presented as 100* ((e^mean variance estimate^)-1)^1/2^.

^b^The relative standard error (RSE) is calculated as 100*(standard deviation/mean value) from 1,046 successful iterations of a non-parametric bootstrap. The 95% confidence interval (95% CI) is displayed as the 2.5 to 97.5 percentiles of the bootstrap estimates.

^c^Post-hoc estimates were calculated as the median and ranges of the empirical Bayes estimates.

*CL*_*ARM*_*/F*: elimination clearance of artemether, *V*_*ARM*_*/F*: apparent volume of distribution of artemether, *CL*_*DHA*_*/F*: elimination clearance of dihydroartemisinin, *V*_*DHA*_*/F*; apparent volume of distribution of dihydroartemisinin, *MTT*; mean transit time, *DUR*; duration of zero order-absorption and F; relative bioavailability. The additive error (σ) variance will essentially be exponential on artithmic scale data. *AUC*: total area under the plasma concentration-time curve after the last dose, C_MAX_: maximum concentration after the last dose and *T*_*MAX*_: Time to maximum concentration.

#### The impact of analysis methodologies

Due to the absence of a non-pregnant control group the results had to be compared to literature. The majority of the pharmacokinetic evaluations of artemether and dihydroartemisinin have been performed using NCA [9,10,42]. The standard procedure of analyzing a metabolite is to adjust the input dose for the metabolite by the relative difference in molecular weight between the parent drug and the metabolite. The metabolite is then assumed to be absorbed from the gut into the systemic circulation. This is inaccurate since the drug in most cases is absorbed as parent drug and then converted to metabolite *in vivo.* The same assumption is made when analyzing the data using a separate pharmacokinetic drug and metabolite model. These approaches might result in non-physiological parameter estimates for the metabolite when analyzing the data both with NCA or separate modeling. A simultaneous pharmacokinetic drug-metabolite model will therefore produce more accurate and physiologically plausible parameter estimates for both the drug and the metabolite.

The observed data were evaluated using NCA and a first-order absorption model followed by a separate one-compartment disposition model for artemether and dihydroartemisinin. Parameter estimates from these approaches were compared to the results obtained using a simultaneous artemether-dihydroartemisinin one-compartment disposition model with first-order absorption to assess the impact of the different pharmacokinetic analysis methodologies. All tested methodologies described the data reasonably well (Table 3). Significant differences in apparent volume of distribution and absorption rate constant were evident when comparing NCA/separate modeling to simultaneous modeling (Table 3). The artemether absorption rate constant was approximately two times higher using simultaneous modeling compared to separate modeling. The artemether apparent volume of distribution obtained with separate modeling was approximately 25% and 50% lower compared to the estimates obtained with NCA and simultaneous modeling, respectively. The effect on the metabolite was even larger with an approximately four times lower estimated apparent volume of distribution for dihydroartemisinin using simultaneous modeling compared to the other two methodologies. This shows clearly that the volume of distribution estimate is affected by the actual absorption model for dihydroartemisinin. Therefore, different modeling approaches will lead to differences in the characterization of both the absorption and the distribution phases for the drug and metabolite.

**Table 3:** Summary of parameter estimates for a comparative analysis of different methodologies

**Parameter**	**Approach 1**	**Approach 2**	**Approach 3**	**P-value**	**P-value**	**P-value**
	**Non compartmental analysis Median [range]**	**Separate modelling Median [range]**	**Simultaneous modelling Median [range]**	**(1 *vs *2)**	**(1 *vs *3)**	**(2 *vs *3)**
Artemether						
CL/F (L/hr)	753 [220-7381]	904 [375-2919]	858 [365-4593]	0.975	0.899	0.972
V/F (L)	1750 [547-11045]	1293 [1279-1301]	2292 [951-4967]	0.002	0.826	0.013
Ka (hr^-1^)	-	0.392 [0.137-2.25]	0.878 [0.381-2.17]	-	-	0.008
AUC_60h-LAST_ (hr × ng/ml)	98.5 [7.24-355]	86.4 [26.5-207]	91.1 [17.4-215]	0.989	0.999	0.983
Dihydroartemisinin						
CL/F (L/hr)	381 [167-1364]	534 [220-1116]	496 [214-1199]	0.311	0.459	0.910
V/F (L)	647 [374-4154]	691 [325-1699]	163 [97-200]	0.247	<0.001	<0.001
Ka (hr^-1^)	-	0.472 [0.472-0.472]	-	-	-	-
AUC_60h-LAST_ (hr × ng/ml)	196 [53.2-449]	140 [67.2-340]	150 [62.2-345]	0.304	0.502	0.930

*CL/F*: elimination clearance, *V/F*: apparent volume of distribution, *Ka*: absorption constant and *AUC*_*60h-LAST*_: total area under the plasma concentration-time curve after the last dose to the last sample time. P-values were presented from an ANOVA test with regression analysis or a student *t*-test (comparing 2 groups) on log transformed parameter estimates. Non-compartmental analysis (Approach 1), separate modelling (Approach 2) and simultaneous modelling (Approach 3) results.

Although the approaches led to significant differences in pharmacokinetic parameter estimates, this may have little clinical relevance. Total exposure of both artemether and dihydroartemisinin were not significantly different for the different approaches. A trend of lower dihydroartemisinin exposure after separate and simultaneous modeling compared to after NCA was observed. This phenomenon resulted from difficulties with fitting the erratic absorption phase.

#### Comparison to literature

Data collected in this study did not allow investigation of auto-induction since patients were sampled only after the last dose. However, a 57% increase in elimination clearance of artemether with each dose (auto-induction) has been suggested in a previous publication [33] and lower artemether exposures were found after multiple dosing [50-53]. The elimination clearance in this study was 4.9-fold higher than that in healthy Pakistani volunteers when sampled after a single dose administration (15.1 L/hr/kg *vs* 3.11 L/h/kg). This could be a result of auto-induction. However, the effect of a different sampling scheme, pregnancy, ethnic differences and/or disease should also be considered [42].

C_MAX_, AUC, T_MAX_, T_1/2_ and CL results obtained by NCA and simultaneous population pharmacokinetic drug metabolite modeling were compared to literature NCA results in Tables 4 and 5. The elimination half-life of artemether (1.96 h) is longer compared to the elimination half-life of dihydroartemisinin (1.39 h), which suggests formation rate limited elimination of dihydroartemisinin. Therefore, the elimination half-life of dihydroartemisinin obtained with compartmental modeling did not reflect its physiological value as a result of flip-flop kinetics. Consequently, the NCA elimination half-life for dihydroartemisinin was considered as the true value.

**Table 4:** A comparison of the artemether pharmacokinetic properties to literature values

			**Artemether**				
		Parameter	AUC (hr × ng/ mL/mg dose)	C_MAX_ (ng/mL/ mg dose)	T_MAX_ (hr)	T_1/2_ (hr)	CL (L/hr/kg)
Pregnant women with uncomplicated malaria	Final model (N = 21)	Median (range)^a^	1.34 (0.203-3.75)	0.411 (0.0930-1.04)	1.16 (0.653-3.81)	1.77 (0.773-2.49)	15.1 (18.6)^b^
	NCA (N = 21)	Median (range)	1.21 (0.09-4.26)	0.443 (0.0710-1.79)	1.27 (0.500-4.00)	1.96 (0.590-4.01)	13.2 (3.21-130)
	NCA in Thai pregnant patients (N = 13) [9]	Median (90% ranges)	0.820 (0.131-3.50)	0.438 (0.175-1.30)	1.00 (0.500-2.00)	1.50 (1.20-7.20)	25.9 (6.00–162)
Non-pregnant patients with uncomplicated malaria	NCA in Thai patients (N = 25) [52]	Mean ± S.D.	2.64 ± 1.36	0.828 ± 0.679	2.0 (1.00-8.00)	2.20 ± 1.00	-
	NCA in Thai patients (N = 13) [54]^c^	Mean (range)	6.03 (3.21-10.23)	1.12 (0.73-1.50)	2.0 (2.0-2.0)	2.6 (1.8-4.7)	3.27
Healthy subjects	NCA in Pakistani subjects (N = 12) [42]^d^	Median (range)	4.53 (1.60-8.30)	2.16 (0.678-4.54)	1.50 (0.500-3.00)	1.88 (1.24-4.00)	3.11 (1.57-11.9)
	NCA in Caucasian subjects (N = 14) [53]	Mean ± S.D.	0.791 ± 0.906	0.343 ± 0.386	1.5 [1–4]	1.6	-
	NCA in Caucasian subjects (N = 8) [50]^e^	Mean ± S.D.	0.350 ± 0.300	0.190 ± 0.130	1.60 ± 0.800	0.500 ± 0.100	-

^a^Median and ranges were derived from the empirical Bayes estimates.

^b^Population estimate (%RSE).

^c^After a single dose of 300 mg artemether and four consecutive 100 mg artemether doses daily.

^d^After a single dose of artemether-lumefantrine (Co-Artem).

^e^After five doses artemether mono-therapy.

*AUC*: area under the concentration-time curve, *C*_*MAX*_: maximum concentration, *T*_*MAX*_: time to maximum concentration and *T*_*1/2*_: elimination half-life. AUC and C_MAX_ were normalized by artemether dose.

**Table 5:** A comparison of the dihydroartemisinin pharmacokinetic properties to literature values

			**Dihydroartemisinin**				
		Parameter	AUC (hr × ng/mL/ mg dose)	C_MAX_ (ng/mL/ mg dose)	T_MAX_ (hr)	T_1/2_ (hr)	CL (L/hr/kg)
Pregnant women with uncomplicated malaria	Final model (N = 21)	Median (range)^a^	2.11 (0.703-5.44)	0.593 (0.185-1.50)	1.37 (0.82-3.89)	0.10 (0.0150-0.470)	8.06 (10.5)^b^
	NCA (N = 21)	Median (range)	2.57 (0.698-5.89)	1.09 (0.247-2.01)	1.83 (0.520-4.00)	1.39 (0.690-2.36)	6.91 (2.29-23.1)
	NCA in Thai pregnant patients (N = 13) [9]	Median (90% ranges)	4.68 (0.391-7.67)	2.16 (0.944-2.94)	1.00 (0.500-2.00)	1.30 (0.900-8.40)	4.50 (2.80–5.40)
Non-pregnant patients with uncomplicated malaria	NCA in Thai male patients (N = 25) [52]	Mean ± S.D.	7.92 ± 3.40	2.69 ± 1.34	2.00 (1.00-6.00)	1.60 ± 0.400	-
	NCA in Thai patients (N = 13) [54]^c^	Mean (range)	11.1 (7.04-15.6)	1.41 (0.871-2.12)	4 (2-4)	3.6 (2.4-4.8)	-
Healthy subjects	NCA in Pakistani subjects (N = 12) [42]^d^	Median (range)	3.97 (2.57-4.74)	1.60 (0.704-2.56)	1.50 (0.750-3.00)	1.78 (1.34-2.20)	3.70 (3.05-5.27)
	NCA in Caucasian subjects (N = 14) [53]	Mean ± S.D.	2.51 ± 1.22	0.982 ± 0.547	1.5 [1-4]	1.5 ± 0.6	-
	NCA in Caucasian subjects (N = 8) [50]^e^	Mean ± S.D.	2.42 ± 0.682	0.818 ± 0.294	1.6 ± 0.8	0.8 ± 0.3	-

^a^Median and ranges were derived from the empirical Bayes estimates.

^b^Population estimate (%RSE).

^c^After a single dose of 300 mg artemether and four consecutive 100 mg artemether doses daily.

^d^ After a single dose of artemether-lumefantrine (Co-Artem).

^e^After five doses artemether mono-theraphy.

AUC: area under the concentration-time curve, *C*_*MAX*_: maximum concentration, *T*_*MAX*_: time to maximum concentration and *T*_*1/2*_: elimination half-life. AUC and C_MAX_ were normalized by dihydroartemisinin dose.

Estimated artemether and dihydroartemisinin exposure in this African pregnant woman population was similar to that reported in pregnant Thai patients [9]. Both the present study and the previously published study in Thai pregnant women [9] showed lower artemether exposures but in a similar range compared to one Thai adult non-pregnant patient population [52]. In contrast, exposures were considerably lower compared to another Thai, adult, non-pregnant patient population [54]. Dihydroartemisinin exposures in both African and Thai pregnant women were lower compared to the two Thai adult non-pregnant patient populations [52,54]. This might suggest a lower artemether and dihydroartemisinin exposure in a pregnant population compared to a non-pregnant patient population. However, this comparison was based on only two available reference populations with different ethnicity [52,54]. Therefore, studies in larger series with non-pregnant control groups are urgently needed to further assess the pharmacokinetics of artemether and dihydroartemisinin in pregnant women.

## Conclusion

In conclusion, the population pharmacokinetic properties of artemether and its metabolite dihydroartemisinin were well described by a simultaneous drug-metabolite model in 21 pregnant women with uncomplicated *P. falciparum* malaria in Uganda. Total exposure of artemether and dihydroartemisinin were somewhat lower in these pregnant women compared to literature adult patient populations. However, these results should be interpreted with caution since ethnicity might have an impact on the pharmacokinetic properties of these drugs. Further studies in larger series with both pregnant and non-pregnant patients are urgently needed to study the pharmacokinetics in this vulnerable group.

## Competing interests

The Wellcome Trust is a UK-based medical research charity and is independent of all drug companies. It has no financial links with the manufacturers of either the diagnostic tests or the drugs used in this study. The authors declared no conflict of interest.

## Authors’ contributions

FN, MD, PG, SM, PP and ET planned and conducted the clinical study. NN, NL performed the drug analysis and FK, JT conducted the pharmacokinetic analysis. FK, JT drafted the manuscript. All authors reviewed the manuscript critically for important intellectual content and approved the final version.
